# Hypercalcemic Encephalopathy as an Initial Presentation of Multiple Myeloma

**DOI:** 10.1155/2020/4746865

**Published:** 2020-02-08

**Authors:** Durga Shankar Meena, Gopal Krishana Bohra, Mahendra Kumar Garg, Abhishek Purohit, Deepak Kumar, Swapnil Tripathi

**Affiliations:** ^1^Department of Medicine, All India Institute of Medical Sciences, Jodhpur, India; ^2^Department of Pathology, All India Institute of Medical Sciences, Jodhpur, India

## Abstract

We report the case of an 84-year-old female presented to us with acute onset altered sensorium. On investigation, neurological and infectious causes were ruled out. On further evaluation, her serum calcium was found elevated (15.07 gm/dl). The diagnosis of hypercalcemic encephalopathy was made with the possibility of multiple myeloma due to raised total protein and globulin levels. Serum electrophoresis, immunofixation, and bone marrow examination confirmed the diagnosis of multiple myeloma. The patient was treated with bortezomib, dexamethasone, and lenalidomide. After 1 week, she improved with normalization of serum calcium. Herein, we highlight hypercalcemia as an important cause of encephalopathy. As our report suggests, metabolic encephalopathy can be the first presentation in multiple myeloma.

## 1. Introduction

Multiple myeloma is characterized by neoplastic proliferation of plasma cells causing increased production of monoclonal immunoglobulin in blood and/or urine. Anemia, bone pain, renal dysfunction, hypercalcemia, fatigue, and weight loss are the common presentation in multiple myeloma [1]. Asymptomatic hypercalcemia is a common metabolic disorder in multiple myeloma. However, hypercalcemic encephalopathy as an initial presentation is rarely reported in the literature. The altered mental status (AMS) in multiple myeloma can be secondary to hypercalcemia, hyperviscosity, uremia, and hyperammonemia [2–5]. Here, we report an 84-year-old female presented in the emergency department with acute onset altered sensorium which on evaluation was diagnosed as hypercalcemic encephalopathy due to multiple myeloma. Our report emphasizes the need for further studies which can help in better understanding of clinical profile and outcome of these patients.

## 2. Case Presentation

**Figure 1:**
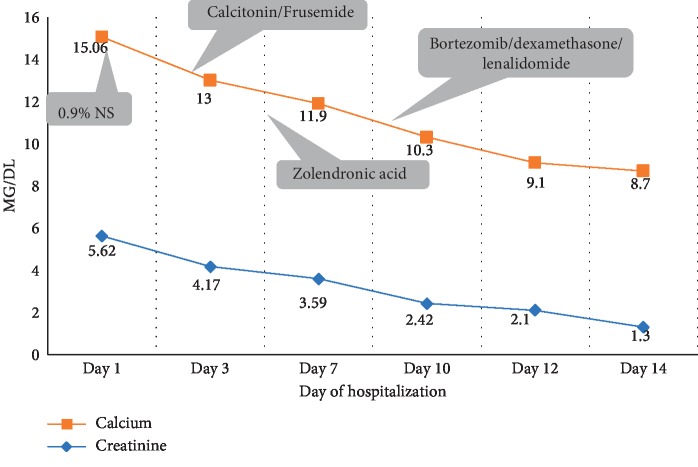
Calcium lowering interventions and improvement in serum calcium and creatinine.

An 84-year-old female presented to the emergency department with complaints of constipation, vomiting, decreased appetite, and excessive urination for the last 10 days and altered sensorium for the last 2 days. There was a history of generalised bone pain for last 3 months for which she was prescribed calcium. Except Bell's palsy 5 months back with complete recovery, past and family history was unremarkable. On examination, the patient was drowsy but arousable. She was dehydrated, and blood pressure was 146/90 mm Hg. Her systemic examination including neurological did not reveal any localisation. Investigations revealed anemia (Hb—6.5 g/dl), deranged renal function (urea—118 mg/dl, creatinine 5.62 mg/dl), increased total protein (9.27 g/dl) and globulin (7.34 g/dl), low albumin (1.93 g/dl), and hypercalcemia (total calcium-15.07 mg/dl). Random plasma glucose (131 mg/dl) and serum electrolytes (sodium/potassium—141/3.92 meq/L) were in the normal range. Serum PTH levels were normal. In addition, serum ammonia levels were also normal (39.4 mmol/L, normal 17–90 mmol/L). Noncontrast computerised tomography of brain showed age-related changes in the bilateral cerebral hemisphere with multiple lytic lesions of varying sizes noticed in the cranial vault and base of the skull. The patient was diagnosed as hypercalcaemic encephalopathy with suspicion of multiple myeloma. To confirm the diagnosis of multiple myeloma, serum protein electrophoresis was performed, which showed M protein (in the beta region) 4.9 g/dL with increased beta 2 microglobulin (9284 ng/ml, normal 609–2366 ng/ml). Bone marrow aspiration showed 60% plasma cells. The final diagnosis of multiple myeloma with hypercalcaemic encephalopathy was made. The patient was treated with iv. fluids (0.9% normal saline, 200 ml/hr with the rate adjusted to maintain urine output 100–150 ml/hr). The patient was also treated with loop diuretics and calcitonin. The next day, zolendronic acid (at modified dose according to GFR) was added. On day 9 of hospitalisation, the patient was put on dexamethasone, lenalidomide, and bortezomib. At the end of 2^nd^ week patient's sensorium was improved, with normalization of serum calcium and renal function (Figure 1).

## 3. Discussion

Altered mental status (AMS) is a vague term that includes various disorders of mental functioning ranges from slight confusion to coma [6]. In elderly patients, altered mental status is the primary cause of admission in the emergency department. Up to 25–30% of the elderly patients present to emergency department with AMS. The etiology of emergency AMS is categorized in primary CNS and nonneurological factors. Studies have reported neurological events (28–37%) as the most common cause of AMS [7] in elderly patients, with systemic and organ failure, infections, and electrolytes derangement being the other common causes. Our patient was admitted with acute onset altered sensorium. The clinical and radiological evaluation did not point to any neurological cause. At the time of admission, the patient had significant renal dysfunction which suggested uremic encephalopathy. However, patient was severely dehydrated, and there were no flapping tremors. This suggested the possibility of prerenal azotemia. Among the toxic metabolic encephalopathies, septic, hepatic, hypo, and hypernatremia are the common causes (Table 1). Her procalcitonin levels were 0.87 ng/ml, which pointed against the possibility of septic encephalopathy. Her initial investigations suggested the possibility of multiple myeloma with hypercalcemic encephalopathy.

AMS in a patient with multiple myeloma can be attributed to uremia, hypercalcemia, hyperviscosity, increased serum ammonia, leptomeningeal myelomatosis (LMM), and intraparenchymal plasmacytoma (Table 1). There are few reports describing altered sensorium as a first presentation of multiple myeloma [5, 8–11]. The common causes of altered sensorium in these reports are direct neurological involvement due to myeloma and uremia (Table 2). Our case is also novel because encephalopathy in our patient was attributed to hypercalcemia. Though our patient had renal dysfunction, it was likely due to prerenal azotemia. Ammonia levels were normal and normal neuroimaging ruled out the possibility of leptomeningeal involvement or plasmacytoma. In our patient, improvement of sensorium was parallel to the normalization of serum calcium, which further consolidated the diagnosis of hypercalcemic encephalopathy.

Acute rise in serum calcium develops neurological symptoms like decreased concentration, confusion, and rarely stupor or coma. Hypercalcemic crisis is a condition characterized by decompensation of hypercalcemia which could be as a first manifestation similar to our case or could have existed for a longer period. The majority of hypercalcemic crisis occurs in primary hyperparathyroidism [12]; however, as our report suggests, decompensate hypercalcemia can present in multiple myeloma. The other common causes of hypercalcemia are malignancies, granulomatous disorders, drugs (lithium, thiazide diuretics, tamoxifen), multiple endocrine neoplasia (MEN), and milk alkali syndrome.

Hypercalcemia is a common metabolic complication in multiple myeloma, occurs in 30% of the patients at some point during the course of disease and 13% of newly diagnosed patients [3]. In multiple myeloma, IL-6, RANKL, macrophage inflammatory protein (MIP) 1a, osteoprotegerin, and IL-3 are important factors contributing to the development of lytic bone disease and hypercalcemia [13]. Hypercalcemia can cause nephrogenic diabetes insipidus which impair the ability to concentrate the urine and produces polyuria and dehydration [14]. In our case, dehydration and prerenal azotemia were responsible for a further fall in GFR, leading to severe hypercalcemia. The worsening of hypercalcemia was attributed to this ongoing vicious cycle of hypercalcemia and dehydration.

In conclusion, altered mental status in an elderly patient can present a significant diagnostic challenge. Emergency physician should consider hypercalcemia as an important cause of metabolic encephalopathy. Prompt workup and intensive medical management to optimize organ function should be prioritized. As our report suggests, in a patient of multiple myeloma, encephalopathy can be the first presentation.

## Figures and Tables

**Table 1:** Differential diagnosis of metabolic encephalopathy and mechanisms in multiple myeloma.

A. Differential diagnosis of acute metabolic encephalopathies	B. Mechanism of encephalopathy in multiple myeloma
1. Septic encephalopathy	1. Hypercalcemia
2. Hepatic encephalopathy	2. Hyperviscosity
3. Uremic encephalopathy	3. Uremia
4. Hyponatremia/hypernatremia	4. Hyperammonemia
5. Hypercalcemia	5. Leptomeningeal myelomatosis (LMM)
6. Hypoglycemia	
7. Hyperosmolar hyperglycemic state (HHS)	
8. Hypoxic-ischemic encephalopathy	
9. Wernicke encephalopathy	

**Table 2:** Clinical, demographic characteristics, treatment, and outcome of reported cases of encephalopathy in multiple myeloma.

Authors; year of publication	Age/gender	Predisposing factors	Treatment	Outcome
Klomp et al. [8]; 2006	60 yr, female	Posterior leucoencephalopathy due to hypercalcemia	Isotonic saline and pamidronate	Improved
Sandhu et al. [9]; 2009	68 yr, male	Paraneoplastic manifestation of multiple myeloma	Intravenous dexamethasone	Complete recovery
Rather et al. [10]; 2014	66 yr, male	Hypernatremia, hypercalcemia azotemia	Bortezumib and dexamethasone	Improved
Sharma et al. [5]; 2015	49 yr, male	Hyperammonemia	Cyclophosphamide, bortezomib, and dexamethasone	Improved
Jaruvongvanich et al. [11]; 2016	39 yr, female	Leptomeningeal myelomatosis and hyperammonemic encephalopathy	Bortezumib and dexamethasone	Developed septic shock, expired due to multisystem organ failure
Current report	84 yr, female	Hypercalcemia	Isotonic saline, zolendronic acid, bortezumib, and dexamethasone	Improved

## References

[1] Kyle R. A., Gertz M. A., Witzig T. E. (2003). Review of 1027 patients with newly diagnosed multiple myeloma. *Mayo Clinic Proceedings*.

[2] Evaldsson U., Ertekin C., Ingvar D. H., Waldenström J. G. (1969). Encephalopathia hypercalcemica. *Journal of Chronic Diseases*.

[3] Sobol U., Stiff P. (2014). Neurologic aspects of plasma cell disorders. *Neurologic Aspects of Systemic Disease Part II*.

[4] Nash E., Ranka P., Tarigopula G., Rashid T. (2015). Primary hyperparathyroidism in pregnancy leading to hypercalcaemic crisis and uraemic encephalopathy. *BMJ Case Reports*.

[5] Sharma S. K., Choudhary D., Handoo A. (2015). An unusual cause of anemia and encephalopathy. *Mediterranean Journal of Hematology and Infectious Diseases*.

[6] Guidotti M., Chiveri L., Mauri M. (2006). Acute encephalopathies. *Neurological Sciences*.

[7] Aslaner M. A., Boz M., Çelik A. (2017). Etiologies and delirium rates of elderly ED patients with acutely altered mental status: a multicenter prospective study. *The American Journal of Emergency Medicine*.

[8] Klomp C. M., Van Den Broek M. W., Buijs J., Beekman R. (2006). Reversible posterior leucoencephalopathy due to hypercalcaemia. *Nederlands tijdschrift voor geneeskunde*.

[9] Sandhu G., Farias A. A., Ranade A., Meisels I. (2009). Altered mental status in a case of multiple myeloma not related to a metabolic cause. *Clinical Kidney Journal*.

[10] Rather P., Hussain M., Bagdadi F. (2014). Localized cutaneous mucinosis associated with multiple myeloma: a rare presentation. *Indian Journal of Dermatology*.

[11] Jaruvongvanich V., Spanuchart I., O-Charoen P., Kitamura C., Sumida L., Roytman M. (2016). An unusual cause of altered mental status in multiple myeloma: an extraosseous manifestation. *Hawaii Journal of Medicine and Public Health*.

[12] Singh D. N., Gupta S. K., Kumari N. (2015). Primary hyperparathyroidism presenting as hypercalcemic crisis: twenty-year experience. *Indian Journal of Endocrinology and Metabolism*.

[13] Oyajobi B. O. (2007). Multiple myeloma/hypercalcemia. *Arthritis Research & Therapy*.

[14] Singla M., Gupta A. (2019). An overlooked cause of polyuria. *The American Journal of Medicine*.

